# LACpG10-HL Functions Effectively in Antibiotic-Free and Healthy Husbandry by Improving the Innate Immunity

**DOI:** 10.3390/ijms231911466

**Published:** 2022-09-28

**Authors:** Weixiong Pan, Zengjue Zhao, Jiahui Wu, Qin Fan, Haobin Huang, Rongxiao He, Haokun Shen, Zitong Zhao, Saixiang Feng, Guanhua Gan, Zhiyang Chen, Miaopeng Ma, Chongjun Sun, Linghua Zhang

**Affiliations:** Guangdong Provincial Key Laboratory of Protein Function and Regulation in Agricultural Organisms, College of Life Sciences, South China Agricultural University, Guangzhou 510642, China

**Keywords:** LACpG10-HL, united agent, antibiotic-free husbandry, innate immunity

## Abstract

Antibiotics are broadly restricted in modern husbandry farming, necessitating the need for efficient and low-cost immunomodulatory preparations in antibiotic-free and healthful farming. As is known to all, CpG oligonucleotides (CpG-ODNs, an effective innate immunostimulatory agent) recognized by TLR9 in mammals (while TLR21 in avians) could collaborate with some united agent to induce stronger immune responses, but the cost is prohibitively expensive for farmers. Here, considering the coordination between TLR2 and TLR9/TLR21, we firstly proposed the idea that the well-fermented *Lactococcus lactis* could be utilized as a CpG-plasmid carrier (LACpG10) to enhance the host’s innate immunity against pathogenic invasion. In the present study, after obtaining LACpG10-HL from homogenized and lyophilized recombinant strain LACpG10, we treated primary chicken lymphocytes, two cell lines (HD11 and IPEC-J2), and chickens with LACpG10-HL, CpG plasmids (pNZ8148-CpG10), and other stimulants, and respectively confirmed the effects by conducting qRT-PCR, bacterial infection assays, and a zoological experiment. Our data showed that LACpG10-HL could induce excellent innate immunity by regulating autophagy reactions, cytokine expression, and motivating PRRs. Interestingly, despite having no direct antiseptic effect, LACpG10-HL improved the antibacterial capacities of lymphocytes and enterocytes at the first line of defense. Most importantly, water-supplied LACpG10-HL treatment reduced the average adverse event rates, demonstrating that LACpG10-HL maintained its excellent immunostimulatory and protective properties under farming conditions. Our research not only contributes to revealing the satisfactory effects of LACpG10-HL but also sheds new light on a cost-effective solution with optimal immune effects in green, antibiotic-free, and healthful husbandry farming.

## 1. Introduction

Antibiotic abuse and misuse in human medicine have resulted in antimicrobial resistance (AMR), leading to the emergence of superbugs. Overuse of antibiotics on livestock contributes to the increased resistance of human pathogens exacerbating the public health crisis [1]. In response to this emerging threat, the European Union (EU) has started AMR monitoring programs and China has implemented strict policies to prohibit or restrict the use of antibiotics in animal husbandry and aquaculture. Antibiotic alternatives are desperately needed to reduce antibiotic use, and boosting innate immunity appears to be an effective strategy.

Strikingly, synthetic oligonucleotides containing unmethylated CpG motifs (CpG-ODNs), which are recognized by Toll-like receptor (TLR) 9 in mammals while by TLR21 in avian and teleost, can precisely meet the demands [2,3,4,5]. Accumulating evidence showed that CpG-ODNs are excellent modulators for innate immune response in protecting hosts of different species from infections [6,7,8,9,10,11,12,13]. Meanwhile, applications of CpG-ODNs as vaccine adjuvants (acquired immunoenhancers) in COVID-19 and Hepatitis B vaccines demonstrated their undeniable safety [14,15,16]. As for chickens especially, Goonewardene et al. reported that CpG-ODNs can endow neonatal chicks with antibacterial immunity, and we have also developed CpG-ODNs as adjuvants for Newcastle disease vaccine in SPF chickens [17,18,19,20,21]. However, we must acknowledge that CpG-ODNs alone is insufficient to adequately protect animals and the high costs limits their broad application in husbandry. Fortunately, suitable CpG-united agents with superior efficacy in curing cancers have been considered by Karapetyan et al. [22], inspiring us to seek CpG-united agents to implement the similar role as antibiotics. To meet the demand for highly efficient production, we selected the CpG plasmids, replicating independently in bacteria, as CpG formulation in this study, whose CpG-ODNs-like performances had been confirmed in our previous study [23].

Lactic acid bacteria (LAB) are well-known probiotics for their competence in hindering the pathogens’ growth, maintaining the balance of intestinal microbiota and enhancing the availability of nutrients, and have been widely used in the food industry and medical applications [24,25]. Recognized by TLR2, LAB can mitigate the development of inflammatory bowel diseases (IBD) and even suppress cancers [24,26,27]. Additionally, specific sequences from the LAB genome, such as AT-ODNs and CpG-ODNs recognized by TLR9, can stimulate innate immunity against lipopolysaccharide (LPS)-induced inflammation [28,29]. Moreover, a recent study reported that utilizing LAB as a carrier successfully delivered therapeutic agents into cancer cells [30]. These findings, together with the well-developed fermentation techniques, indicate that LAB would be safe, commercial, and efficient carriers for CpG plasmids delivery.

Inspiringly, the proven synergistic connection between TLR2 and TLR9 suggests that LAB can be more than a carrier. Sørensen et al. reported that TLR2 and TLR9 functioning together can awaken the first line of host defense against *herpes simplex* virus infection in the brain [31]. Similarly, Tursi et al. found that TLR2 and TLR9 worked coordinately to mediate immune responses against bacterial curli-DNA complexes [32]. Adequate evidence showed that TLR2 and TLR9 can synergistically induce the immune responses in mouse macrophages and human cells [33]. Additionally, TLR21 is homologous to TLR9 and has the same innate immunostimulatory activity as TLR9 in avian and teleost [5,34]. Based on the above findings, we hypothesized that the utilization of CpG plasmids plus LAB could induce stronger immune responses in livestock, making it a preferable strategy in antibiotic-free farming.

Initially, this study constructed recombinant plasmids containing CpG motifs, which were subsequently transformed into *L. lactis* NZ9000 to attain the recombinant strain LACpG10. Later, after homogenization followed by lyophilization, the lyophilized powder of LACpG10 homogenate was obtained, which is referred to hereafter as “LACpG10-HL”. In vitro, the immunoenhancing effects and related mechanisms of LACpG10-HL were investigated respectively in chicken lymphocytes, IPEC-J2 and HD11. Ultimately, LACpG10-HL was fed to chickens orally to confirm the efficiency of LACpG10-LH under farming conditions. In summary, this study firstly proposed the idea that *L. lactis* could be utilized as a carrier and a cost-effective united agent of CpG-DNA to improve the therapeutic efficacies of CpG-DNA, and consequently provides a promising solution in the post-antibiotic era in the green, healthy, and sustainable development of husbandry.

## 2. Results

### 2.1. Construction of Recombinant pNZ8148-CpG10 and Generation of LACpG10-HL

To construct the recombinant pNZ8148-CpG10, the CpG10 fragment was cloned into the plasmid pNZ8148 between the restriction sites *Sac* I and *Xba* I. After confirming by sanger sequencing, we obtained the recombinant pNZ8148-CpG10 (Figure S2).

Subsequently, pNZ8148-CpG10 were electroporated into *L. lactis* NZ9000 to generate the recombinant strain NZ9000-pNZ8148-CpG10 (LACpG10). Afterward, we homogenized the LACpG10 cells and then implemented lyophilization to attain the lyophilized powder of LACpG10 homogenates (LACpG10-HL) as our CpG agents for further analyses (as shown in experimental designs (Figure S1)).

### 2.2. Assessment of LACpG10-HL on Cellular Level

#### 2.2.1. LACpG10-HL Can Induce Strong Immunity in Chicken Lymphocytes

**Figure 1:**
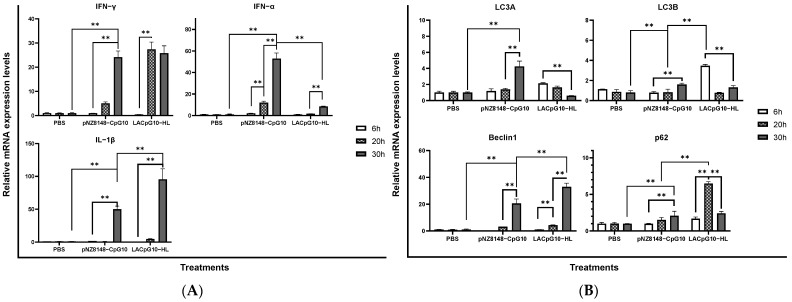

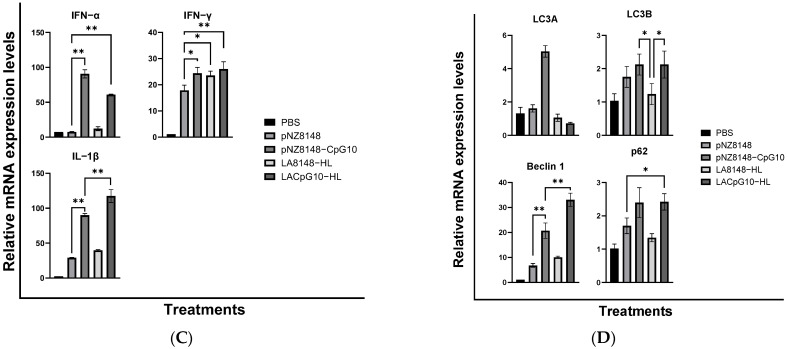
Immunostimulatory effects of LACpG10-HL on chicken lymphocytes. Chicken lymphocytes were stimulated by PBS, pNZ8148-CpG10, and LACpG10-HL at different time points to detect the relative mRNA level of cytokines (**A**) and autophagy proteins (**B**). Afterward, two more control stimulants, including pNZ8148 and LA8148-HL, were added to further confirm the efficacies of LACpG10-HL at 30 h (**C**,**D**). These data were presented as the relative expression ratio with normalization to β-actin. Each column is represented with mean ± SD from three repeated experiments. Significant differences between different treatments were indicated by “*” (* *p* < 0.05, ** *p* < 0.01).

Based on our previous studies, we have found that chicken lymphocytes were suitable for the preliminary evaluation of CpG-DNA [35]. Therefore, in this section, we stimulated chicken lymphocytes with pNZ8148-CpG10 and LACpG10-HL, respectively, to determine the expression level of genes related to innate immunity (IL-1β, IFN-γ, and IFN-α, Figure 1A and LC3A, LC3B, Beclin 1, and p62, Figure 1B) at different time points (6 h, 20 h, and 30 h).

Our results showed that the cytokines and autophagy levels were both upregulated by the two CpG agents after a 30 h co-culture. Interestingly, compared with the plasmids alone, LACpG10-HL showed a higher expression level of IL-1β while less IFN-α, for the contained immune stimulus such as teichoic-acid and various proteins might change the immunoregulatory pattern [36]. Additionally, the IFN-γ peak among the three timepoints of LACpG10-HL treatment reached 20 h, which was 10 h earlier than pNZ8148-CpG10 (Figure 1A). As for genes related to autophagy, the two treatments showed distinct enhancement profiles (Figure 1B). Generally, we found that LACpG10-HL treatments led to the earlier expression of LC3A, LC3B, Beclin 1, and p62 compared to pNZ8148-CpG10, indicating that LACpG10-HL has an advantage over pNZ8148-CpG10 in terms of rapidly enhancing immune responses. Moreover, the better immunostimulatory effects of LACpG10-HL were further confirmed at 30 h (vs. pNZ8148-CpG10 and LA8148-HL, Figure 1C,D). In summary, LACpG10-HL had favorable immunostimulatory efficacies in inducing innate immunity in chicken lymphocytes.

#### 2.2.2. LACpG10-HL Can Effectively Motivate the Pattern Recognition Receptors (PRRs)

**Figure 2:**
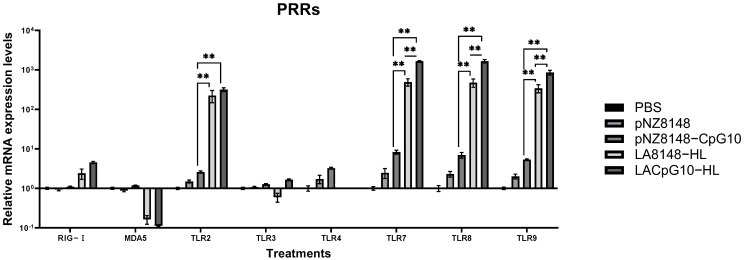
LACpG10-HL effectively motivates the pattern reignition receptors (PRRs). IPEC-J2 cells were respectively treated with PBS, pNZ8148, pNZ8148-CpG10, LA8148-HL, and LACpG10-HL for 30 h. Afterward, the cells were collected for qRT-PCR. The relative mRNA expression levels of PRRs were detected to show how LACpG10-HL initiated the sensors for invading pathogens. Columns represent the means of 3 repeats for each treatment. Significant differences between different treatments were indicated by “**” (** *p* < 0.01).

In order to further clarify the antibacterial potentiality of LACpG10-HL, we detected the gene expressions of PRRs [37], including RLRs (RIG-Ⅰ and MDA5), as well as TLRs (TLR2, TLR3, TLR4, TLR7, TLR8, and TLR9) in IPEC-J2 (a model cell of intestines [38]) stimulated by CpG agents and control stimulants. LACpG10-HL upregulated the expression level of RIG-Ⅰ compared to pNZ8148-CpG10 and LA8148-HL (Figure 2). However, LACpG10-HL greatly downregulated the expression of MDA5 (vs. pNZ8148-CpG10 and LA8148-HL, Figure 2). In an aspect of TLRs, upregulations of TLR2, TLR7, TLR8, and TLR9 transcripts were observed in every treatment, with the exception that TLR3 transcript was downregulated in LA8148-HL treatment while upregulated in pNZ8148-CpG10 and LACpG10-HL treatments (Figure 2). Additionally, the TLR4 transcript was undetectable in LA8148-HL and LACpG10-HL treatments.

#### 2.2.3. Immunostimulatory Effects of LACpG10-HL against Intestinal Pathogens

In order to evaluate the effects of LACpG10-HL in infectious circumstances, *Pseudomonas aeruginosa*, *Shigella sonnei*, Enterohemorrhagic *Escherichia coli* (EHEC), and *Streptococcus pyogenes* [35] were used to challenge the cell models (HD11 and IPEC-J2), mimicking the natural surroundings.

##### pNZ8148-CpG10 and LACpG10-HL Have No Direct Antibacterial Effects against Intestinal Pathogens

**Figure 3:**
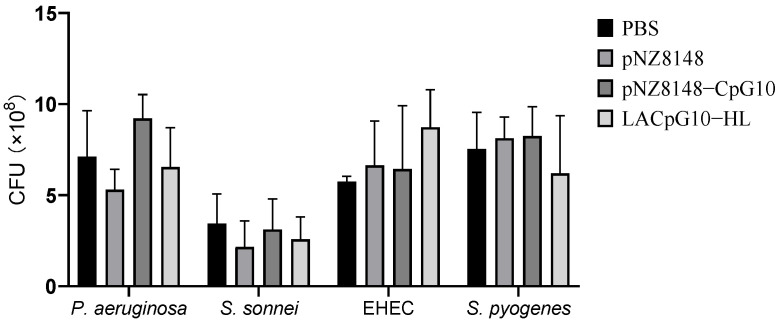
LACpG10-HL possessed no direct antibacterial effects. After 24 h co-culturing, the four pathogens, including *P. aeruginosa*, *S. sonnei*, EHEC, and *S. pyogenes,* with the stimulants, the CFU of pathogens indicated that LACpG10-HL or other stimulants, compared to PBS treatment, had no negative effects on the growth of the pathogens. Columns represent the means of 3 repeats for each treatment. Error bars represent the Standard deviation (SD).

Before being co-culture with cells, we evaluated the effects of each sample on the growth of *P. aeruginosa*, *S. sonnei*, EHEC, and *S. pyogenes* to detect whether they had direct antibacterial effects. As results are shown in Figure 3, there was no significant difference between the four treatments for either pathogenic bacterium. On average, the colony-forming units (CFU) of the four bacteria with the four treatments were pretty close, indicating that CpG agents had no direct effects on the suppression of the bacteria growth. That is, the presence of living cells is required for pNZ8148-CpG10 and LACpG10-HL to perform their immunostimulatory function.

##### LACpG10-HL Can Simultaneously Boost Antibacterial Immunity 

**Figure 4:**
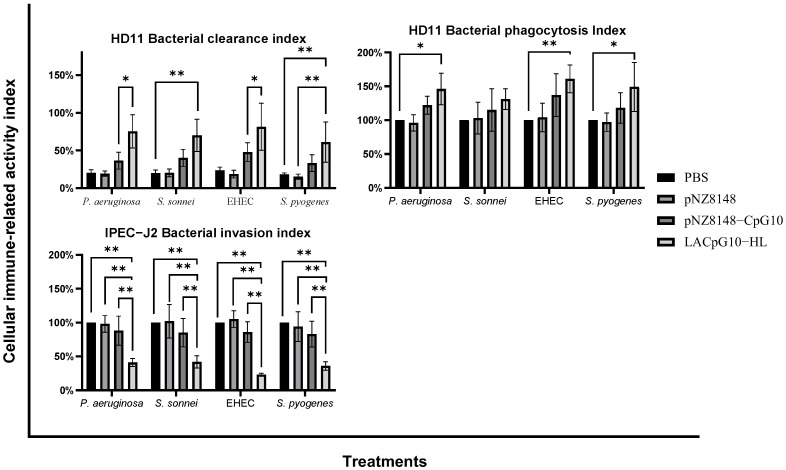
LACpG10-HL enhanced the antibacterial capacities of macrophages and enterocytes. HD11 and IPEC-J2 cells were pretreated with the stimulants for 30 h. Afterward, HD11 cells were co-cultured with pathogens at MOI = 1 and MOI = 10 to detect the corresponding bacterial clearance index and bacterial phagocytosis index. Meanwhile, IPEC-J2 cells were co-cultured with pathogens at MOI = 10 to detect the bacterial invasion index. Each column is represented with mean ± SD from three repeated experiments. Significant differences between different stimulants under infection of the same pathogen were indicated by “*” (* *p* < 0.05, ** *p* < 0.01).

Afterward, we examined the role of LACpG10-HL in defending pathogenic infections on intestinal cellularity. Firstly, we investigated the influence of LACpG10-HL treatment on the bacterial clearance capacity of the avian macrophage HD11. By viewing the HD11 bacterial clearance index (Figure 4), we found that PBS and pNZ8148 showed no efficacies on these macrophages, while both pNZ8148-CpG10 and LACpG10-HL showed antibacterial capability and the latter one was more prominent. By comparison, we inferred that LACpG10-HL could strongly stimulate HD11 to kill pathogens, suggesting that LACpG10-HL would be a better choice on the side of improving the bacterial clearance in macrophages.

Additionally, we investigated whether LACpG10-HL could effectively enhance the capacity of HD11 for phagocytosing pathogens. Therefore, we calculated the bacterial phagocytosis index in HD11 infected with pathogens at MOI = 10. Like the tendency of the HD11 bacterial clearance index, compared to the other groups in the HD11 bacterial phagocytosis index (Figure 4), LACpG10-HL upregulated the bacterial phagocytosis of HD11, manifesting that LACpG10-HL could better mobilize the macrophages to phagocytose invasive bacteria, and consequently directly kill the bacteria at the host first defense.

Meanwhile, we also detected the effect of LACpG10-HL on the bacterial invasion of IPEC-J2. IPEC-J2, an intestinal enterocyte, is the initial barrier against pathogens in the intestine, which triggers us to uncover whether LACpG10-HL can better protect enterocytes from pathogens. Contrary to the former indices, the lower this IPEC-J2 bacterial invasion index, the stronger the ability of cells to resist external infection, as well as the enhancement of innate immunity [39]. As the results show (Figure 4), both pNZ8148-CpG10 and LACpG10-HL downregulated the invasion of the four pathogens into IPEC-J2, while LACpG10-HL showed more significant results (*p* < 0.01 vs. the other treatments). This observation revealed that LACpG10-HL better protected enterocytes from pathogens averting the direct damage to the intestine barrier, consistent with the HD11 results.

**Figure 5:**
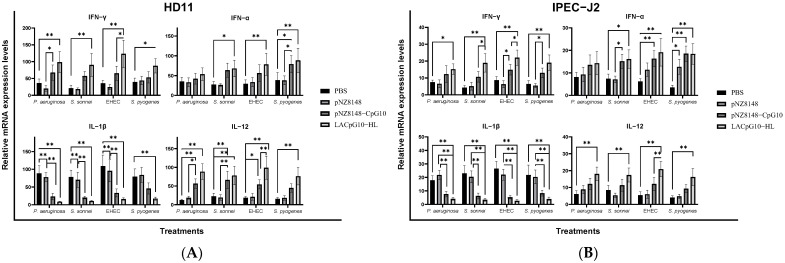
LACpG10-HL boosted antibacterial immunity in HD11 and IPEC-J2. The two cells were infected with the pathogens at MOI = 10 to detect the relative mRNA expression levels of cytokine genes. (**A**) presented the results in HD11 and (**B**) presented those in IPEC-J2. The reference gene of mRNA detection was β-actin. Each column is represented with mean ± SD from three repeated experiments. Significant differences between different stimulants under infection of same pathogen were indicated by “*” (* *p* < 0.05, ** *p* < 0.01).

To further validate the observed phenomenon in this section, we determined the expression level of genes related to CpG-induced immunity (IFN-γ, IFN-α, IL-1β, and IL-12, Figure 5) and qPCR results echoed the above results (Figure 5). Compared to PBS or pNZ8148 group, pNZ8148-CpG10 and LACpG10-HL effectively upregulated the immune responses (IFN-γ, IFN-α, and IL-12) against bacteria in both cell types while decreasing the level of inflammation cytokine (IL-1β). In summary of the entire Figure 5, LACpG10-HL was much more efficient in enhancing antibacterial immunity than pNZ8148-CpG10 alone.

### 2.3. Assessment of LACpG10-HL on Individual Level under Farming Conditions

Based on the results of the cytological level experiments, to further assess the effects of LACpG10-HL in daily farming, we conducted a zoological experiment on a chicken farm and consequently obtained ideal results. To simulate a real-world farming scenario, we directly added LACpG10-HL to the food or drinking water every day for 14 days with triple repeats in each group. After corresponding treatments, clinical signs and morbidity were recorded daily for 16 days. As results shown (Table 1), compared to PBS, the pNZ8148-CpG10 and food additive or water-supplied LACpG10-HL, respectively, reduced the average adverse event rates by 50%, 75%, and 90%, indicating the notably protective effects of LACpG10-HL under normal farming conditions.

## 3. Discussion

Our laboratory confirmed that CpG agents (CpG motifs in ODN and plasmids) are excellent immunostimulants but whose production cost is prohibitively expensive for widespread animal farming use [13,40,41]. To overcome this defect, we came up with the probiotic LAB as a CpG-plasmid carrier whose fermentation cost is controllable. Based on our previous findings and the coordinated functions between TLR2 and TLR9/TLR21 [3,33], we were interested in further excavating the cooperated immunostimulatory activity of CpG plus LAB (LAB containing CpG plasmids) in chickens. In this study, we managed to obtain LACpG10-HL and investigate its immunostimulatory efficacies and related mechanism on the cellular and individual levels under farming conditions. Ultimately, we hoped to disclose its capacity to boost the host’s innate immunity in antibiotic-free farming.

To function in a similar role as antibiotics, we firstly assessed the direct anti-infectious capacity of the CpG plasmids or LACpG10-HL alone. We treated them to the four pathogens, including *P. aeruginosa*, *S. sonnei*, EHEC, and *S. pyogenes*, and found that they had no direct inhibitory effect on the pathogens, implying that the antibacterial role of pNZ8148-CpG10 or LACpG10-HL is primarily dependent on the immunoregulatory capacities.

Therefore, the gene expressions of active molecules involved in innate immunity were elevated. Initially, we chose to look at the transcriptional responses of the related genes in primary chicken lymphocytes treated with the stimulants. Autophagy is a conserved catabolic process in all eukaryotic cell types related to lysosomal activity implicating in the direct elimination of microbes, control of inflammation, antigen presentation, lymphocyte homeostasis, and secretion of immune mediators [42,43]. Macroautophagy, the main fashion of autophagy in lymphocytes, is initiated by the Beclin 1 composed complex and functions through the activities of LC3 and p62 [44]. CpG ODN has been reported to act as an infectious signal to trigger antitumor and antibacterial autophagy by activating TLR9 [45,46], thus lending credence to the evaluation of the LACpG10-HL efficacies by detecting alterations of autophagy-related gene expressions. Indeed, according to our experimental data, we were convinced that pNZ8148-CpG10 induced autophagy in chicken lymphocytes by upregulating Beclin 1, LC3A, LC3B, and p62, while expectative higher expressions were achieved with LACpG10-HL treatment. 

On the other hand, since bacterial infection is one of the classic instigators of inflammation [47], we took innate immune-related genes (IFN-α, IFN-γ, and IL-1β) stimulated by LACpG10-HL into account in this study. Among the detected cytokines, IFN-α mainly possesses antiviral activity [48,49], while its antibacterial activity against *Neisseria gonorrhoeae* and *Streptococcus* has also been reported [50,51]. Moreover, IFN-γ, mainly secreted by Th1 cells and natural killer cells, implicates innate immune responses [52,53], and IL-1β is a proinflammatory cytokine involved in the activation of macrophages and epithelial cells [54,55]. By treating the chicken lymphocytes with LACpG10-HL, we obtained upregulated expressions of these cytokines, further confirming that LACpG10-HL exerted an immunostimulatory effect on the lymphocytes, consistent with our previous findings [23,56]. 

To go a step further into the capacities of LACpG10-HL against infection and circumvent the defect that chicken lymphocytes are not ideal model cells for bacterial infection, we turned to the two cell lines (HD11 and IPEC-J2) with infections of four pathogenic bacteria to mimic the infectious conditions [56]. Under the infectious pattern, we found that three of the cytokines, including IFN-α, IFN-γ, and IL-12, were upregulated, and most of the regulations were significant compared with the control group, whereas the downregulated expression of IL-1β was also observed. Since IFN-γ and IL-12 are Th1 cytokines acting in the Th1 immune responses, which is beneficial for preventing infections [54,57], our results displaying the upregulated level of these cytokines were in correspondence with the above data of bacterial invasion, phagocytosis, and clearance. Moreover, our former findings reported that the sustained upregulation of IFN-γ was advantageous to bacterial clearance [11,23]. Furthermore, in animals, IL-12 can activate the natural killer cells to produce IFN-γ, while in return, IFN-γ can activate macrophages to produce IL-12, so a self-amplifying loop is formed [58,59]. The higher expression of IL-12 in this study indicated that LACpG10-HL could boost the loop to induce Th1 immune responses. As for the results of IL-1β, they guided us to our previous studies, which revealed that IL-1β was upregulated by our stimulant at the early stage of infection, but it was downregulated to maintain the normal inflammatory level after the invaded bacteria were cleared [10,11]. Notably, combining the lower bacterial invasion with the experimental results of cytokines in IPEC-J2, we believe that LACpG10-HL has an immunoprotective effect on intestinal enterocytes, the first line of anti-infectious defense against invaded bacteria [60,61,62]. 

The mechanism behind the stronger immunostimulatory efficacies of LACpG10-HL is comprehensible. Firstly, based on the direct proof of the homologous relationship and similar functional effects among TLR21 and TLR9 [34,63], as well as the synergistic roles of TLR2 and TLR9 [31,32,33], we believe that there is an underlying synergism between TLR2 and TLR21, which explains the functional mechanism of LACpG10-HL. Secondly, abundant studies had illustrated that CpG-ODNs and AT motifs within the LAB genome were both immunostimulatory [64,65], which were released during homogenization of LACpG10-HL and increased the categories and dosages of CpG-DNA, consequently leading to the stronger immune responses. Ultimately, the other compositions of LAB are contributors to the enhanced activities. Polysaccharides derived from LAB have been reported to have the protective property of delivering probiotics against dehydration and harmful surroundings [66], indicating that the same effect may occur in the delivery of pNZ8148-CpG10. Moreover, for the other derivatives from LAB, the teichoic acids stimulate TLR2-dependent proinflammatory cytokine production, while the cell wall proteins are reported to be immunoregulatory [36], which preserve the probiotic property of LAB and further advance the LACpG10-HL efficacies.

Thus, the upstream sensors that trigger the expressions of the tested cytokines and autophagy proteins need to be detected. We tried to investigate the LACpG10-HL’s influences on PRRs. PRRs are a broad range of molecules, including viral sensors (RIG-Ⅰ, MDA5, TLR3, TLR7, and TLR8), LPS sensor (TLR4), LAB sensor (TLR2), and CpG sensor (TLR9 in mammalian/ TLR21 in avian), that the organisms use to sense the invading pathogens, thereby initiate the anti-infectious immunity [37,67]. Specific CpG ODNs partially implemented their antiviral activity through RIG-Ⅰ and increased the expression of IFNs [68]. LAB could negatively modulate the inflammatory function of TLR4 [26,69]. Chuang et al. reported that avian TLR21, homologous to TLR9 in mammals, can be activated by a broad range of CpG-DNA sequences [34]. In this study, LACpG10-HL treatment significantly upregulated the expression levels of TLR2 and TLR9 (TLR21 in avians), supporting our previous hypothesis of the coordinated function between TLR2 and TLR21. Moreover, LACpG10-HL treatment also upregulated RIG-Ⅰ, TLR3, TLR7, and TLR8 transcripts, showing that LACpG10-HL had a global antiviral efficacy. Whereas the downregulated expressions of MDA5 and TLR4 suggested that LACpG10-HL had a particular preference for antimicrobial immunity. These, plus the downregulated IL-1β expression in this study, indicated that LACpG10-HL could induce a controlled inflammatory response while keeping its fine immunostimulatory efficacy.

As is known to all, the daily farming environment is not aseptic nor avirulent. Therefore, to evaluate the efficacy of LACpG10-HL more exactly in animals, we conducted a zoological experiment on chickens under a normal farming scenario, where chickens may face different pathogens, including viruses. The much lower adverse event rates with LACpG10-HL treatment validated that the combination of NZ9000 components and pNZ8148-CpG10 had a stronger capacity in initiating the host innate immunity against pathogens under farming conditions, according to the cellular data including cytokines, autophagy proteins, and PRRs, plus the bacterial clearance, phagocytosis, and invasion indexes. At the same time, the better efficacy of the drinking water-supplied group suggested that utilizing LACpG10-HL delivered by drinking water could be a better method to reduce the immune functional loss in destructive conditions such as stomach acid, thus improving the effect of LACpG10-HL. Notably, the high cost of CpG-DNA has greatly restricted its broadly commercial utilization in farming, herein *L. lactic* with CpG plasmids, produced from cost-effective fermentation [70], has shown ideal immunomodulatory efficacies in avian, fitting in solving the defect of CpG-DNA utilization and widely spreading to husbandry farming.

The initial purpose of our study was to excavate the role of CpG plus LAB (LACpG10-HL) in green, antibiotic-free, and healthful husbandry. If it works, LACpG10-HL will provide an effective approach to help animals defend against invading pathogens without antibiotics utilization. At this point, we have reached our goal, but it should be noted that this study overall was to evaluate the healthful breeding serviceability of LACpG10-HL in vivo and in vitro. However, determining the molecular mechanisms underlying protective immunity elicited by LACpG10-HL has been challenging. Further investigation will be necessary to reveal how the LAB components boost the CpG-DNA efficacy and the interaction between the PRRs and involve distinct signal pathways triggered by LACpG10-HL, which we plan to go a further step into. Notwithstanding its limitation, in our research, we confirmed that LACpG10-HL indeed enhanced the antibacterial immunity and globally displayed clinical protections in organisms, developing a new solution in antibiotic-free and healthful husbandry farming.

In conclusion, our study demonstrated that CpG plus LAB (LACpG10-HL) outperformed CpG-DNA alone in eliciting innate immunity, and LACpG10-HL showed satisfactory protections against pathogens in vivo and in vitro, showing that LAB could be utilized as an efficient CpG-united agent. The primary roles of LACpG10-HL were to motivate the initial sensors (PRRs) for pathogens and thus elicit corresponding immune responses, including cytokine expressions and antibacterial autophagy. Our demonstrations that LACpG10-HL had better immunostimulatory effects and opened the door to exploring a cost-effective solution with ideal immune outcomes in antibiotic-free and healthy husbandry farming.

## 4. Materials and Methods

As shown in Figure S1, the experimental design of the whole study would be divided into five parts: (1) the construction of recombinant plasmids and corresponding *L. lactis* strains and the preparation of the CpG agents and control stimulants; (2) the assessment of immunoenhancing effects of the stimulants in chicken lymphocytes and IPEC-J2; (3) detections of direct antibacterial activity of the stimulants on four pathogens; (4) determinations of indexes related to antibacterial immunity in IPEC-J2 and HD11; (5) healthful farming evaluations of the stimulants.

### 4.1. Chemicals and Materials

SanPrep endotoxin-free plasmid extraction kit and SanPrep Column Plasmid Mini-Preps Kit were purchased from Sangon Biotech, Shanghai, China. Lymphocyte Separate Kit was purchased from Haoyang, Tianjin, China. RNAiso Plus was acquired from TaKaRa, Beijing, China. BeyoRT™ II First-Strand cDNA Synthesis Kit (RNase H minus) was purchased from Beyotime, Shanghai, China. 2 × TSINGKE^®^ Master qPCR Mix was acquired from Tsingke, Beijing, China. The enzymes and kits for biological operations were purchased from Takara (Beijing, China) and Tsingke (Beijing, China). Cell culture reagents were obtained from Gibco (Guangzhou, China).

### 4.2. Plasmids, Organisms, and Culture Conditions 

The plasmid pNZ8148 was stored in our laboratory (Table S1). Recombinant plasmid pNZ8148-CpG10 was constructed through the insertion of CpG10 [23] from the pre-synthesized pUC19-CpG10 into the *Xba* I and *Sac* I restriction sites of pNZ8148 (Figure S2). The two plasmids, pNZ8148 and pNZ8148-CpG10, were transformed into *E. coli* MC1061 for amplification. Gene synthesis and sequencing were performed by Genewiz (Guangzhou, China). Afterward, the plasmids were extracted from MC1061 with a SanPrep endotoxin-free plasmid extraction kit (Sangon Biotech, Shanghai, China). Followed by electroporation, we constructed the recombinant strains LA8148 and LACpG10. Recombinant strains were cultivated in 200 mL of GM17 medium (M17 medium with 0.5% glucose) containing 10 µg/mL of chloramphenicol for 18 h. After that, bacteria were centrifugal resuspended in 10 mL of sterile water and homogenized at 4 °C and 1000 bar three times before freeze-dried. Additionally, according to Yao et al. protocol [71], we pretreated LA8148 and LACpG10 with lysozyme for 10 min and afterward removed the lysozyme by centrifugation and washing twice with 5% glucose solution. Subsequently, the plasmids within LA8148 and LACpG10 were extracted with SanPrep Column Plasmid Mini-Preps Kit (Sangon Biotech, Shanghai, China) and double-checked the concentrations of plasmids by Micro Drop (Denley, Guangdong, China) and Agarose Gel Electrophoresis. Ultimately, we obtained LACpG10-HL and LA8148-HL, which were stored at −20 °C. Before use, LACpG10-HL and LA8148-HL were respectively dissolved in 4 mL PBS to get the corresponding 10× working solution with the final plasmid concentration of 10 µg/mL in corresponding strains. The enzymes and kits for biological operations were purchased from Takara (Beijing, China) or Tsingke (Beijing, China).

In this study, six kinds of bacteria, including *Lactococcus lactis* subsp. Cremoris NZ9000, Escherichia coli MC1061, *Pseudomonas aeruginosa* ATCC 27853 (*P. aeruginosa*), *Shigella sonnei* CMCC 51592 (*S. sonnei*), Enterohemorrhagic *Escherichia coli* ATCC 35150 (EHEC) and *Streptococcus pyogenes* ATCC 19615 (*S. pyogenes*) [23], were preserved in our laboratory (Table S1). Moreover, three kinds of cells were used in this study, including HD11, IPEC-J2, and chicken lymphocytes (Table S1). The chicken lymphocytes were isolated from fresh chicken spleen using the Lymphocyte Separate Kit (Haoyang, Tianjin, China) [23].

Unless noted, cell culture conditions are the same as follows: NZ9000 and the recombinant strains were inoculated in GM17 medium (M17 medium with 0.5% glucose) at 30 °C, while the rest bacteria were cultivated in Luria–Bertani (LB) medium at 37 °C. The chicken lymphocytes were diluted in a complete RPMI-1640 medium supplemented with 10% fetal bovine serum (FBS) and antibiotics (100 U penicillin/mL and 100 μg streptomycin/mL) to a final concentration of 5.0 × 10^6^ cells/mL after isolation. Then, 1 mL of the cell suspension was added to each well of 24-well plates and cultured at 41 °C under 5% CO_2_. HD11 cells were maintained under the same conditions as the former. Moreover, IPEC-J2 cells were maintained in DMEM-F12 (1:1) with 10% FBS and 5% CO_2_ at 37 °C. Cell culture reagents were obtained from Gibco (Guangzhou, China).

### 4.3. Assessment of Immunostimulatory Effects with Chicken Lymphocytes and IPEC-J2

In this section, three cytology experiments were performed to assess the immunoenhancing effects of the stimulants on cells under conditions without pathogens. Based on the perspective of in vitro cytology, the immunostimulatory functions of various plasmids and CpG agents were evaluated. Treatments for each experiment are as follows:

Experiment 1: Initially, to determine the optimal stimulation time, we directly added three stimulants to the chicken lymphocytes. Grouping: (1) PBS, (2) pNZ8148-CpG10 (10 μg/mL), and (3) LACpG10-HL solution (containing 10 μg/mL pNZ8148-CpG10). Afterward, the cell culture plates of chicken lymphocytes were placed under suitable conditions for 6 h, 20 h, and 30 h. Ultimately, cells were collected by centrifugation at 2000 rpm for 3 min and preserved at −80 °C for later quantitative real-time PCR (qRT-PCR) (Section 4.6) to detect the expression of genes encoding IFN-γ, IFN-α, IL-1β, LC3A, LC3B, Beclin 1, and p62 [23,72]. 

Experiment 2: Additionally, two more control stimulants, including pNZ8148 and LA8148-HL, were added to confirm the immunostimulatory capacities. Grouping: (1) PBS, (2) pNZ8148 (10 μg/mL), (3) pNZ8148-CpG10 (10 μg/mL), (4) LA8148-HL solution (containing 10 μg/mL pNZ8148), and (5) LACpG10-HL solution (containing 10 μg/mL pNZ8148-CpG10). Except that the stimulation time was the best (30 h) determined by Experiment 1, the other experimental operations and detections were the same as in Experiment 1.

Experiment 3: To further evaluate the immunostimulatory effects of the CpG agents, the IPEC-J2 cells treated with the stimulants were taken into account to detect the expression of genes encoding pattern recognition receptors (PRRs) including RIG-Ⅰ and MDA5 (RIG-I-like receptors, RLRs), as well as TLR2, TLR4, TLR7, TLR8, and TLR9 (Toll-like receptors, TLRs) [37]. IPEC-J2 cells were adjusted to 1.0 × 10^6^ cells/mL in a complete RPMI-1640 medium, and the cell suspension was added to a 6-well cell culture plate at 1 mL per well. The details of grouping were the same as described in Experiment 2. After treatments, IPEC-J2 cells were inoculated for 30 h, collected by centrifugation at 2000 rpm for 3 min, and preserved at −80 °C for later qRT-PCR (Section 4.6). 

### 4.4. Detections of Antibacterial Activity

Antibacterial activities of the stimulants were determined by quantifying the colony-forming unit (CFU) of pathogens after 24 h co-culture. Four pathogens, including *P. aeruginosa*, *S. sonnei*, EHEC, and *S. pyogenes* (Table S1), were treated with PBS, pNZ8148 (10 μg/mL), pNZ8148-CpG10 (10 μg/mL), and LACpG10-HL solution (containing 10 μg/mL pNZ8148-CpG10) for 24 h with CFU counted [73].

### 4.5. Determinations of Cytokine Expressions and Indexes Related to Antibacterial Immunoactivities 

In this section, we focused on the antibacterial immunoactivities of the stimulants in HD11 and IPEC-J2 cells infected by pathogens. Before adding pathogens, HD11 and IPEC-J2 cells were seeded into 24-plates at 1 × 10^5^ cells/well without antibiotics [35]. When the cell adherence rate reached 75–85% after 8–12 h, the cells were pretreated respectively with PBS, pNZ8148 (10 μg/mL), pNZ8148-CpG10 (10 μg/mL), and LACpG10-HL solution (containing 10 μg/mL pNZ8148-CpG10) for 30 h. Meanwhile, the four pathogens described in Section 4.2 were cultured overnight, then collected and washed with PBS three times and adjusted to 1 × 10^8^ CFU/mL in DMEM-F12 (1:1) (Gibico, Guangzhou, China). After the pretreatments, we conducted four infection experiments on the two cell lines to determine three indexes (including the bacterial clearance index, the phagocytosis index, and the bacterial invasion index) and the relative mRNA expressions of IL-12, IFN-γ, IFN-α, and IL-1β. Detailed treatments for each experiment are described as follows:

Experiment 1: Evaluations of bacterial clearance in HD11.

Pretreated HD11 cells were co-cultured with four pathogens at MOI (multiplicity of infection, amount of microbe/number of infected cells) = 1 for 2 h. To calculate the bacterial clearance index, the infected HD11 cells were lysed with 0.5% deoxycholate. Then, the cells were washed and lysed in H₂O containing 0.1% Triton X-100. Ultimately, the bacteria were resuspended in PBS, and serial dilutions of this lysate were plated on LB agar for counting CFU. The bacterial clearance index was expressed in percentages using the following formulation: (CFU counts in stimulants-treated infected cells/CFU counts in PBS-treated infected cells) × 100 [39].

Experiment 2: Evaluations of bacterial phagocytosis in HD11.

Pretreated HD11 cells were co-cultured with four pathogens at MOI = 10 for 1 h. The infected IPEC-J2 cells were washed with PBS after harvesting and incubated for 30 min in media containing 20 ug/mL gentamicin (Gibco, Guangzhou, China) to kill extracellular bacteria. Then, the washed cells were lysed with 0.5% deoxycholate and lysis in H₂O containing 0.1% Triton X-100. The number of bacteria in lysates was determined by counting CFU on an LB agar plate. The bacterial phagocytosis index was expressed in percentages using the following formulation: (CFU counts in stimulants-treated infected cells/CFU counts in PBS-treated infected cells) × 100 [74].

Experiment 3: Evaluations of bacterial invasion in IPEC-J2.

Except that HD11 was replaced by IPEC-J2, and the other experimental operations were the same as described in Experiment 2 in Section 4.5. The formulation of the bacterial invasion index was expressed as follows: (CFU counts in stimulants-treated infected cells/CFU counts in PBS-treated infected cells) × 100.

Experiment 4: Detections of the relative mRNA expressions.

Infected HD11 and IPEC-J2 cells in the previous experiments were also collected after corresponding co-cultures and washed three times with sterile PBS, by then stored at −80 °C to detect the relative mRNA expressions of IL-12, IFN-γ, IFN-α, and IL-1β with qRT-PCR (Section 4.6).

### 4.6. Quantitative Real-Time PCR (qRT-PCR) 

RNA extraction and cDNA synthesis were used to test the relative mRNA level of immune-related genes of the samples collected in Section 4.3 and Section 4.5 [10]. Briefly, total RNA was extracted using RNAiso Plus (TaKaRa, Beijing, China) according to the manufacturer’s protocol. Moreover, the first-strand cDNA was synthesized by using BeyoRT™ II First-Strand cDNA Synthesis Kit (RNase H minus) (Beyotime, Shanghai, China) according to the manufacturer’s protocol.

qRT-PCR was performed on CFX96 real-time system (BioRad, Hercules, CA, USA) with 2 × TSINGKE^®^ Master qPCR Mix (Tsingke) following the manufacturer’s protocol using the primers as shown in Table S2. In qRT-PCR, the genes were normalized to the β-actin, and the relative expression of each gene was calculated by using the 2^−ΔΔCt^ method. The primers used in qRT-PCR were synthesized by Tsingke. All the samples were performed in triplicate, and results were recorded in mean ± standard deviation [35].

### 4.7. Animal and Treatments 

Fast yellow chicken [75] was used to evaluate the efficacy of the stimulants on the individual level on a chicken farm. All chickens were from the Guangdong Huanong Zhengda Poultry Co., Ltd. These chickens (30 days old) were randomly divided into four groups and three replicates for each group. To match the real farming scenario, we add the stimulants directly to the feed or drinking water every day for 14 days. The detailed grouping was described as follows: (1) 1 mL PBS in feed (PBS group, N (Number) = 33, 33, 34). (2) 10 μg pNZ8148-CpG10 in feed (pNZ8148-CpG10 group, N = 8, 8, 9). (3) 100 μL LACpG10-HL in feed (food additive LACpG10-HL group, N = 31, 31, 32). (4) 100 μL LACpG10-HL in drinking water (water-supplied LACpG10-HL group, N = 37, 37, 38).

Clinical signs and morbidity were examined daily for 16 days post immunization. The adverse event rates were determined by observations of each bird’s signs of drowsiness and huddling, runny nose, difficulty breathing, diarrhea, or any other morbidity [76,77,78]. 

All the experimental procedures were approved by the Institutional Animal Care and Use Committees (IACUC) of South China Agricultural University (SCAU), Guangzhou city, Guangdong province, China. No specific permits were required for this study described here. The study area is not privately owned or protected in any way, and the field of studies did not involve endangered or protected species.

### 4.8. Statistical Analyses

All experiments were performed at least three repetitions, and the results are expressed as mean ± standard error of the mean (SEM). The data in this study are conducted by one-way analysis of variance (ANOVA) and Student’s t-test for independent samples in GraphPad Prism 8 software. All the data charts are drawn by GraphPad Prism 8 software. One and two symbols above the histogram represents a significant difference (*p*-value < 0.05) and an extremely significant difference (*p*-value < 0.01).

## Figures and Tables

**Table 1:** Adverse event rates under normal farming conditions.

Group	Number of Individuals with Clinical Signs			Average Adverse Event Rates (%)
PBS	6/33	8/33	4/34	17.9
pNZ8148-CpG10	1/8	1/8	0/9	8.3
Food additive LACpG10-HL	1/31	2/31	1/32	4.2
Water-supplied LACpG10-HL	1/37	0/37	1/38	1.8

## Data Availability

All data are included in the text and Supplementary Materials.

## Supplementary Materials

The following supporting information can be downloaded at: https://www.mdpi.com/article/10.3390/ijms231911466/s1.

## Abbreviations

CpG-ODNs	synthetic oligonucleotides containing unmethylated CpG motifs.
LACpG10	NZ9000 with pNZ8148-CpG10.
LA8148	NZ9000 with pNZ8148.
LACpG10-HL	the lyophilized powder of LACpG10 homogenate.
LA8148-HL	the lyophilized powder of LA8148 homogenate.
AMR	antimicrobial resistance.
EU	European Union.
LAB	lactic acid bacteria.
SFP	specific pathogen free.
IBD	inflammatory bowel diseases.
LPS	lipopolysaccharide.
PRRs	pattern reignition receptors.
RLRs	RIG-I-like receptors.
CFU	colony-forming units.
MOI	multiplicity of infection, amount of microbe/number of infected cells.
