# Supplementary material for: LACpG10-HL Functions Effectively in Antibiotic-Free and Healthy Husbandry by Improving the Innate Immunity
Source: Int J Mol Sci. 2022 Sep 28;23(19):11466. doi: 10.3390/ijms231911466 (PMC9569488; doi:10.3390/ijms231911466)
Supplement: Supplementary file 1 [file ijms-23-11466-s001.zip › ijms-1888047-supplementary.pdf]

International Journal of
Molecular Sciences

MDPI

Article

LACpG10-HL Functions Effectively in Antibiotic-Free and Healthy Husbandry by Improving the Innate Immunity

Weixiong Pan †, Zengjue Zhao †, Jiahui Wu, Qin Fan, Haobin Huang, Rongxiao He, Haokun Shen, Zitong Zhao, Saixiang Feng, Guanhua Gan, Zhiyang Chen, Miaopeng Ma, Chongjun Sun and Linghua Zhang *

Guangdong Provincial Key Laboratory of Protein Function and Regulation in Agricultural Organisms, College of Life Sciences, South China Agricultural University, Guangzhou 510642, China
* Correspondence: lhzhang@scau.edu.cn
† These authors contributed equally to this work.

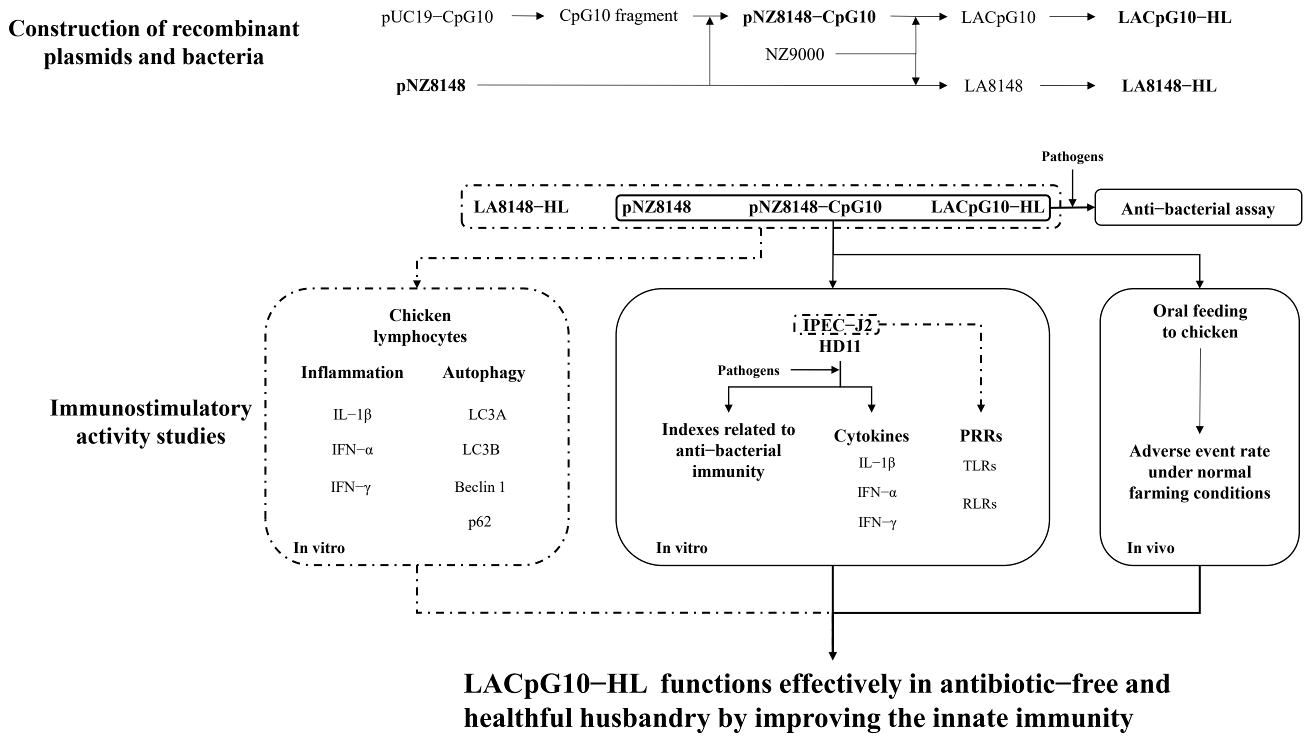

Figure S1. Design flow chart for the entire study.

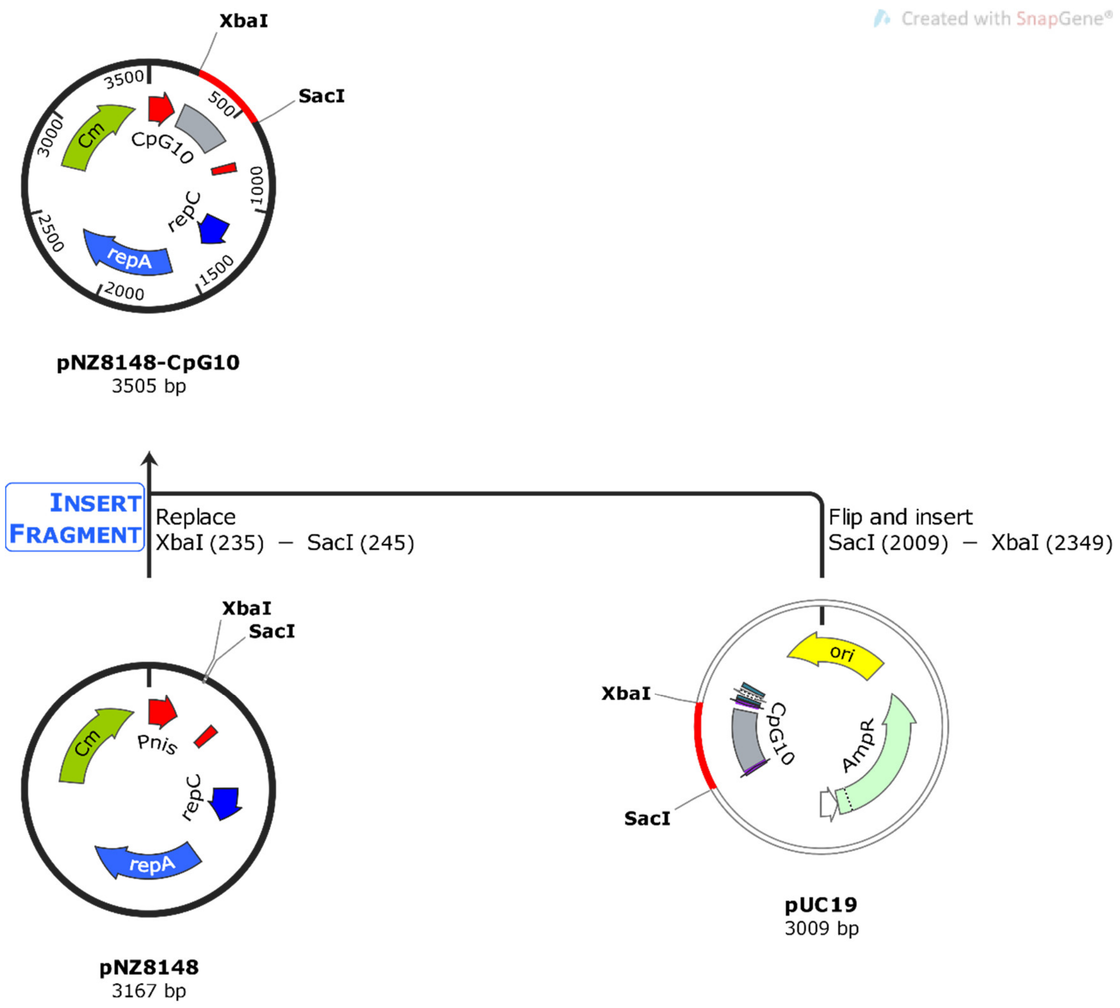

Figure S2. Construction of recombinant plasmid.

The CpG10 fragment was pre–synthesized and cloned into pUC19. pNZ8148 and pUC19–CpG10 were double digested with *Sac*I and *Xba*I, and the needed fragments (pNZ8148 backbone and CpG10 fragment) were purified from an agarose gel. Next, the fragments fused by the ligation reaction and transformed into DH5α to get the correct transformants. In the end, colony PCR and sanger sequencing were performed for confirmation.

Table S1. Plasmids and strains used in this study.

Names	Characteristics	Reference or source
**Plasmids**		
pNZ8148	*L. lactis'* high copy number expression vector	Lab stock
pUC19–CpG10	pUC19 containing synthesized CpG10 fragment	This study
pNZ8148–CpG10	pNZ8148 containing synthesized CpG10 fragment	This study
**Stains**		
Lactococcus lactis NZ9000		Lab stock
LA8148	NZ9000 with pNZ8148	This study

LACpG10	NZ9000 with pNZ8148−CpG10	This study
Escherichia coli MG1655	Cloning strain of pNZ8148	Lab stock
Pseudomonas aeruginosa ATCC 27853		Lab stock
Shigella sonnei CMCC 51592		Lab stock
Enterohemorrhagic *Escherichia coli* ATCC 35150		Lab stock
Streptococcus pyogenes ATCC 19615		Lab stock
Chicken lymphocytes	Lymphocytes isolated from fresh chicken spleen	This study
HD11	Macrophage−like chicken cell line	Lab stock
IPEC−J2	Intestinal porcine enterocytes isolated from the jejunum of a neonatal unbuckled piglet	Lab stock

Table S2. Primers used for quantitative real−time PCR (qRT−PCR) assays.

Primer	Sequence	Product size (bp)	Gene ID
GalIFN−γ−F	ACAAGTCAAAGCCGCACATCAAAC	86	NM_205149.2
GalIFN−γ−R	TTTCACCTTCTTCACGCCATCAGG		
GalIFN−α−F	CCAGCACCTCGAGCAAT	133	XM_046936231.1
GalIFN−α−R	GGCGCTGTAATCGTTGTCT		
GalIL−1β−F	TCGGGTTGGTTGGTGATG	244	XM_046931582.1
GalIL−1β−R	TGGGCATCAAGGGCTACA		
GalLC3A−F	TTACACCCATATCAGATTCTTG	143	XM_040688401.2
GalLC3A−R	ATTCCAACCTGTCCCTCA		
GalLC3B−F	AGTGAAGTGTAGCAGGATGA	193	NM_001031461.2
GalLC3B−R	AAGCCTTGTGAACGAGAT		
GalBeclin 1−F	CGACTGGAGCAGGAAGAAG	115	NM_001006332.1
GalBeclin 1−R	TCTGAGCATAACGCATCTGG		
Galp62−F	GACCCAGCCAAGACTACCAT	240	XM_040682727.2
Galp62−R	CAGAGGCATGTAGTTTCGGC		
Galβ−actin−F	TCAGGTCATCACCATTGGC	81	NM_001006332.1
Galβ−actin−R	CAGGACTCCATACCCAAGAAAG		
SusIFN−γ−F	GGCCATTCAAAGGAGCATGGATGT	149	NM_213948.1
SusIFN−γ−R	TGAGTTCACTGATGGCTTTGCGCT		
SusIFN−α−F	CATCTGCAAGGTTCCCAATGGC	207	NM_214393.1
SusIFN−α−R	CATGAGGGGATCCAAAGTCCC		
SusIL−1β−F	GCTAACTACGGTGACAACAATAATG	186	NM_214055.1
SusIL−1β−R	CTTCTCCACTGCCACGATGA		
SusIL12−F	TCTGAGCCGGTCACAACTGC	296	NM_214013.1
SusIL12−R	AGGCGCTGTGCTCCTGACAC		

Susβ−actin−F	CACGCCATCCTGCGTCTGGA	380	XM_021086047.1
Susβ−actin−R	AGCACCGTGTTGGCGTAGAG		
SusDDX588−F(RIG−I)	AGTAAACTGCACCCCAAACCA	189	NM_213804
SusDDX588−R(RIG−I)	GGCATGCACGGTCTGAACTC		
SusIFIH1−F (MDA5)	GAAATGATGCACTTGCCCGC	132	NM_001100194
SusIFIH1−R (MDA5)	GACTTGGCTGATCTGTGGCTA		
SusTLR2−F	TGACACCGCCATCCTCATTCTG	209	NM_213761
SusTLR2−R	AGACCAGCATCGGACCAAGAC		
SusTLR3−F	ACATCCAGCTGTACAAAACC	122	NM_001097444
SusTLR3−R	AAGCCAAGCAAAGGAATCAT		
SusTLR4−F	TGACAACATCCCCACATCAGT	188	NM_001113039
SusTLR4−R	TTCCCGTCAGTATCAAGGTGG		
SusTLR7−F	AGACAAGCACTTGACAGCGA	102	NM_001097434
SusTLR7−R	TGGAAGGAGGCTGGAGTGAT		
SusTLR8−F	CACGCAAAGACCACCACCAACT	278	XM_021079509
SusTLR8−R	TGAGCCAGGGCAGCCAACATA		
SusTLR9−F	CTCTGGTCCAACTGCCTATGC	288	NM_213958
SusTLR9−R	TCCGAGACAGGTCCAAGGTGAA		
